# Susceptibility pattern of *Salmonella enterica* against commonly prescribed antibiotics, to febrile-pediatric cases, in low-income countries

**DOI:** 10.1186/s12887-021-02497-3

**Published:** 2021-01-15

**Authors:** Priyatam Khadka, Januka Thapaliya, Shovana Thapa

**Affiliations:** 1 Medical Microbiology, Tri-Chandra Multiple Campus, Kathmandu, Nepal; 2 International Friendship Children’s Hospital, Kathmandu, Nepal

**Keywords:** Enteric fever, Low-income countries, Nepal, Pediatric, *Salmonella enterica*

## Abstract

**Background:**

In most low-income countries, febrile-pediatric-cases are often treated empirically with accessible antibiotics without periodic epidemiological surveillance, susceptibility testing, or minimal lethal dose calculations. With this backdrop, the study was undertaken to evaluate the susceptibility trend of *Salmonella enterica* against the commonly prescribed antibiotics.

**Methods:**

All isolates of *Salmonella enterica* were identified by standard protocols of biotyping and serotyping, then tested against antibiotics by the modified Kirby disk-diffusion method. Minimum Inhibitory Concentration (MIC) of isolates was determined by the agar-dilution method and compared with disk diffusion results and on nalidixic-acid sensitive/resistant strains.

**Results:**

Among 1815 febrile-pediatric patients, 90(4.9%) isolates of *Salmonella enterica* [serovar: *Salmonella* Typhi 62(68.8%) and *Salmonella* Paratyphi A 28(31.1%)] were recovered. The incidence of infection was higher among males, age groups 5 to 9, and patients enrolling in the out-patient department (OPD). On the disk-diffusion test, most isolates were sensitive against first-line drugs i.e.cephalosporins, and macrolides. However, against quinolones, a huge percentile 93.3%, of isolates were resistant [including 58 Typhiand 26 Paratyphiserovar] while nearly 14% were resistant against fluoroquinolones.

When MICs breakpoint were adjusted as follows: 4 μg/ml for azithromycin, ≥1 μg/ml for ciprofloxacin, 2 μg/ml for ofloxacin, 8 μg/ml for nalidixic acid, and 1 μg/ml for cefixime, higher sensitivity and specificity achieved. Compared to other tested antibiotics, a low rate of azithromycin resistance was observed. Nevertheless, higher resistance against fluoroquinolones was observed on NARS strain.

**Conclusion:**

Higher susceptibility of *Salmonella enterica* to the conventional anti-typhoidal drugs (amoxicillin, chloramphenicol, cotrimoxazole, cephotaxime) advocates for its reconsideration. Although, the lower susceptibility against fluoroquinolones among nalidixic-acid-resistant Salmonella (NARS) strain negates its empirical use among the study age group.

**Supplementary Information:**

The online version contains supplementary material available at 10.1186/s12887-021-02497-3.

## Background

Enteric fever, a serious bloodstream infection caused by *Salmonella enterica* species, is a burgeoning global threat—disproportionately affecting more than 17 million people with recorded mortality 178,000,000, annually [1–3]. The infection prevails irrespective of any age categories; nevertheless, children of low and middle-income countries have the highest documented burden [4–6]. Also, the most MDR (multi-drug resistant)strains(H58 strain) had recovered from these countries [7, 8]. Therefore, the condition is excruciating, the people of these regions had to face the dual challenge of economic constraints and obstinate MDR strain. In Nepal, the MDR H58 *Salmonella* Typhi(S. Typhi) seems to have been substituted by non-MDR H58 carrying the S83F mutation in gyrA and other mutations—associated with reduced susceptibility to fluoroquinolones [9, 10]. Likewise, *Salmonella* Paratyphi A(S.Paratyphi) frequently carries fluoroquinolone non-susceptibility alleles in gyrA and parC; however, none of these isolates were MDR [11, 12]. Nevertheless, the possible transmissibility of MDR H58 could not be neglected since many cases of MDR H58 had been reported from the abutting countries: India, Pakistan, and Bangladesh [13, 14]. This background justifies the rationale of our study.

The infection is primarily treated by antimicrobial therapy; nonetheless, is becoming difficult due to changes in the susceptibility trend of the pathogen. Ironically, without a periodic epidemiological survey, susceptibility testing, and minimal lethal dose calculations; children in developing countries are still being treated empirically or even self-medicated with accessible antibiotics [15]. These could be the reasons, why there is significant morbidity and mortality in most developing countries [3, 16, 17]. Therefore, in these countries, where distinct diagnostic and therapeutic modalities could not be assessed due to economic constraints, evaluating antimicrobial susceptibility pattern of the isolate against commonly prescribed antibiotics is crucial for clinical management.

## Methods

### Study design and sample population

A cross-sectional study was conducted over one year (April 2017–March 2018) in International Children Friendship Hospital (ICFH), a tertiary care hospital for children, in Kathmandu, Nepal. Febrile-pediatric subjects (up to 14 years of age) with clinical suspicion of enteric fever were enrolled, in our study. The clinical investigation/suspicion to enteric fever was made by the respective unit pediatrician, relying upon the clinical history and most occurring clinical presentations—high-grade fever, abdominal pain, sore throat, loss of appetite, and generalized weakness. A pre-tested questionnaire was administered to each patient or from their guardian to ascertain demographic characteristics, symptoms, brief clinical history, and history of antibiotic use (if under therapy). Furthermore, commonly prescribed antibiotics (either from clinicians or in self-medicated cases) for enteric fever, in the record of local pharmacies, were evaluated. Data regarding personal information (patient’s demographic including residence locality) and existing other infectious diseases were coded and kept confidential.

### Inclusion and exclusion criteria

All febrile-pediatric patients with clinical suspicion of enteric fever were enrolled; however, only *Salmonella enterica* recovered from blood samples were studied. The other isolates, nonetheless, obtained from the sample (other than blood) after a period, and from the same patient were considered as duplicated isolates, hence excluded.

### Laboratory methods

The blood samples were collected aseptically (about 2-3 ml) and cultured in brain heart infusion broth (HiMedia, India) as per guidelines set by the American Society for Microbiology (ASM) for conventional blood culture [18]. Further, isolation and identification of the isolates were done by standard microbiological techniques—biotyping (colony morphology, staining reaction, and biochemical characteristics) and serotyping using specific antisera (Denka Seiken Co. Ltd., Tokyo, Japan) [18]. The samples were considered sterile if no bacterial growth was observed on the sub-culture after 7 days of aerobic incubation at 37 °C.

### Antimicrobial susceptibility testing

The antimicrobial susceptibility of *Salmonella enterica* against antibiotics was tested by the disk diffusion method [modified Kirby-Bauer method] on Mueller Hinton agar (Hi-Media, India) in compliance with standard procedures recommended by the Clinical and Laboratory Standards Institute (CLSI), Wayne, PA, USA [19]. The antimicrobials tested were: amoxicillin (10 μg), azithromycin (15 *μ*g), cefixime (5 *μ*g), ceftriaxone (30 *μ*g), cephotaxime (30 *μ*g), chloramphenicol (30 *μ*g), ciprofloxacin (5 *μ*g), cotrimoxazole (25 *μ*g), nalidixic-acid (30 *μ*g), ofloxacin (5 *μ*g). The interpretation of susceptibility results was made based on interpretative zone diameters suggested by CLSI. For the standardization of susceptibility testing, *Escherichia coli* ATCC (American Type Culture Collection) 25,922 and *Staphylococcus aureus* ATCC 25923 were used as control organisms.

### Determination of minimum inhibitory concentrations (MICs)

Only, MICs of most prescribed antibiotics—recommended by clinicians, and self-medicated cases—recorded in the local pharmacies were done. MICs of ciprofloxacin, ofloxacin, nalidixic-acid, azithromycin, and cefixime were determined by agar dilution method as suggested by Andrews [20] based on CLSI guidelines [19]; and were classified sensitive or resistant accordingly. Of the total 90 isolates, MICs value of only 71 isolates were determined by the agar dilution method since 9 of the isolates were not preserved and 10 isolates could not be revived.

### Comparison between disk-diffusion test and MICs

The results of disk-diffusion test and agar dilution test of the most commonly prescribed antibiotics (azithromycin, cefixime, ciprofloxacin, ofloxacin, and nalidixic-acid) were compared by WHONET 5.4 software.

### Correlation between NARS and fluoroquinolones (FQs)

The obtained isolates were broadly classified into nalidixic-acid sensitive Salmonella (NARS) strains and nalidixic-acid sensitive Salmonella (NASS) strains and correlated against the resistance pattern of FQs.

### Data management and analysis

The data obtained was entered in Microsoft Office Excel 2007 and analyzed by Statistical Package for Social Sciences (SPSS) version 16.0. The susceptibility data (with observed zone size) and MIC values of ciprofloxacin, ofloxacin, nalidixic-acid, azithromycin, and cefixime were analyzed by WHONET 5.4 software.

## Result

### Patients’ demographics

During the study period, a total of 1815 febrile-pediatric patients, suspected to have enteric fever (including 997 male and 818 female patients) were enrolled. Of the total study population, the culture positivity rate was higher in males 55 (5.5%) compared to females 35 (4.2%). Similarly, the percentage was higher among the patient of age-group (5 to 9 years) and those from out-patient department (OPD). Before attending to hospital, 304 (218 on ciprofloxacin; 86 ofloxacin) had a self-medicated history, they visited our hospital when there was no symptomatic resolution (Table 1).

**Table 1:** Patients’ demographics

Patients demographics	Total suspected	Confirmed (%)	***Salmonella enterica*** serovar	
			Typhi (%)	Paratyphi (%)
**Gender**				
Male	997	55 (5.5)	39 (70.9)	16 (29.1)
Female	818	35 (4.2)	23 (65.7)	12 (34.3)
**Age group**				
< 1 year	389	8 (2.05)	5 (62.5)	3 (37.5)
1 to 4 years	452	17 (3.76)	11 (64.7)	6 (35.2)
5 to 9 years	439	37 (8.42)	27 (72.9)	10 (27.1)
10 to 14 years	485	28 (5.77)	19 (67.8)	9 (32.2)
**Patients distribution**				
Out-patient	1389	73 (5.2)	53 (72.6)	20 (27.4)
In-patient	426	17 (3.9)	12 (70.59)	5 (29.41)
**Self-medicated history**				
Ciprofloxacin therapy	218	6 (2.7)	5 (83.3)	1 (16.7)
Ofloxacin therapy	86	4 (4.6)	3 (75.0)	1 (25.0)

### Bacterial isolates

Of total samples, 4.9% (*n* = 90) *Salmonella enterica* isolates were recovered, among them 62(68.8%) were S. Typhi and the remaining 28 (31.1%) were S.Paratyphi.

### Antibiogram of *Salmonellaenterica* isolates on disk-diffusion test

On susceptibility testing, most of the recovered isolates were sensitive to first-line antibiotics (amoxycillin, chloramphenicol, and cotrimoxazole), third-generation cephalosporins (cephotaxime, ceftriaxone, and cefixime), and macrolides (azithromycin). However, to FQs, 93.3% of the isolate were quinolone-resistant and nearly 15% were resistant to ciprofloxacin and ofloxacin (Table 2).

**Table 2:** Antimicrobial susceptibility pattern of *Salmonella enterica* on disk diffusion test

Antibiotics Group	Antibiotic used	Antibiotic susceptibility					
		Susceptible		Intermediate		Resistant	
		n	%	n	%	n	%
penicillin	amoxycillin	90	100	0	0	0	0
phenicols	chloramphenicol	90	100	0	0	0	0
synthetic	cotrimoxazole	90	100	0	0	0	0
cephalosporins	cephotaxime	90	100	0	0	0	0
	ceftriaxone	89	98.9	1	1.1	0	0
	cefixime	89	98.9	1	1.1	0	0
macrolides	azithromycin	88	97.8	2	2.2	0	0
fluoroquinolones	ofloxacin	70	77.7	7	7.8	13	14.4
	ciprofloxacin	65	72.2	13	14.4	12	13.3
	nalidixic-acid	6	6.7	0	0	84	93.3

### Antibiogram of *Salmonella enterica* based upon MICs

Susceptibility results of 71 isolates were tested for MIC of antibiotics: ciprofloxacin, ofloxacin, nalidixic-acid, cefixime, and azithromycin are shown in Table 3. The sensitive/resistant isolates were classified according to the CLSI guideline based upon the MICs of antibiotics.

**Table 3:** Antimicrobial susceptibility pattern of *Salmonella enterica* based upon MICs

Antibiotics Group	Antibiotic used	Antibiotic susceptibility			
		Susceptible		Resistant	
		Number	%	Number	%
macrolides	azithromycin	70	98.6	1	1.4
cephalosporins	cefixime	68	98.8	3	4.2
fluoroquinolones	ciprofloxacin	49	69	22	31
	ofloxacin	49	69	22	31
	nalidixic-acid	13	18.3	58	81.7

### Ciprofloxacin MICs versus disk diffusion test

41 isolates with MICs ≥0.5 μg/ml had ZOI (zone of inhibition) ≤ 28 mm; 7 isolates with MICs ≥1 μg/ml and 1 isolate with MIC of ≥2 μg/ml had ZOI 22 mm; among 22 isolates with MICs ≤8 μg/ml,10 isolates had ZOI ≤17 mm while the remaining had ZOI ≤15 mm.

### Ofloxacin MICs versus disk diffusion test

36 isolates with MICs ≤1 μg/ml had ZOI ≥ 17 mm except one which had ZOI 12 mm;13 isolates with MICs of ≤4 μg/ml had ZOI 17 mm; of 22 isolates with MICs ≥8 μg/ml, 19 isolates had ZOI ≤14 mm while the remaining are with 17 mm ZOI.

### Nalidixic acid MICs versus disk diffusion test

58 isolates with MICs ≥32 μg/ml had ZOI ≤11 mm; among 13 isolates with MICs 16 μg/ml, 9 had ZOI ≥19 mm while the remaining had ZOI 13 mm.

### Cefixime MICs versus disk diffusion test

44 isolates with MICs ≤0.5 μg/ml had ZOI ≥ 19 mm; 24 isolates with MICs ≥1 μg/ml and ≤ 8 μg/ml had ZOI 19 mm; of the remaining 3 isolates with MICs ≥32 μg/ml,1 isolate had ZOI 17 mm while the other 2 isolates had ZOI 19 mm.

### Azithromycin MICs versus disk diffusion test

67 isolates with MICs ≤8 μg/ml had ZOI 19 mm; 3 isolates with MICs 16 μg/ml had ZOI 18 mm while the remaining single isolate with MICs 64 μg/ml had ZOI 17 mm.

**Fig. 1:**
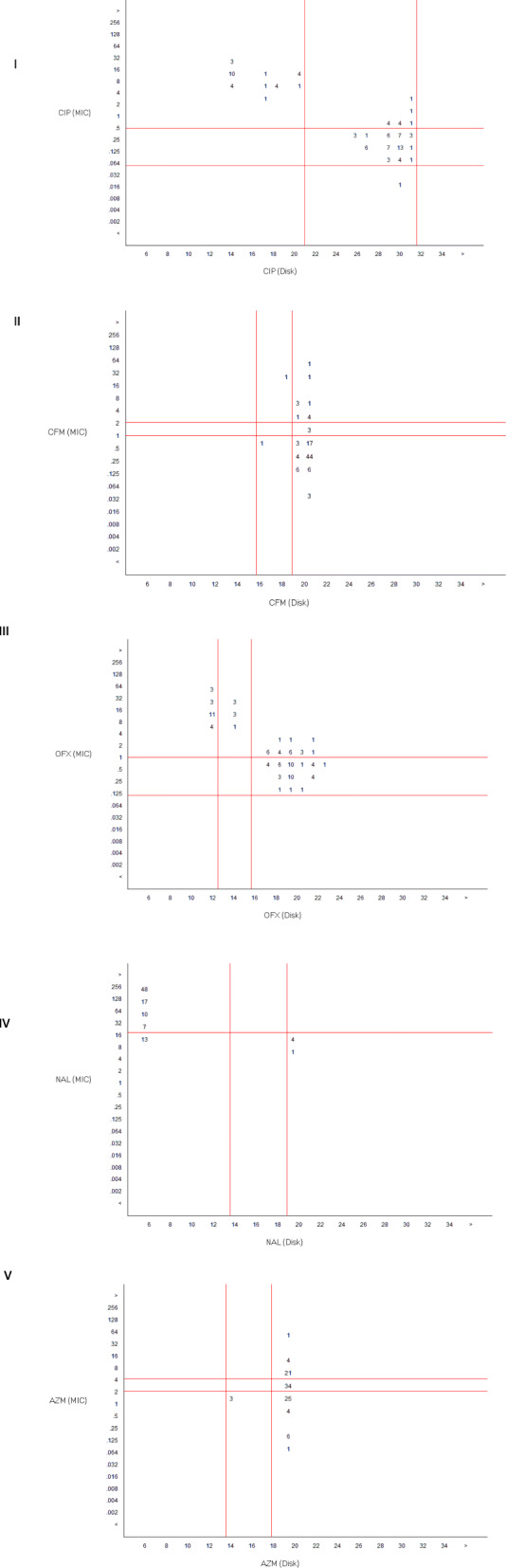
Scatter plot relating (I) ciprofloxacin (II) cefixime (III) ofloxacin (IV) nalidixic acid (V) azithromycin MICs to zone of inhibition diameter from respective antibiotic disk

The comparison of MIC and disk diffusion of these antibiotics is shown in Fig. 1.

Relying upon these results, MIC breakpoint was adjusted 4 μg/ml for azithromycin, ≥1 μg/ml for ciprofloxacin, 2 μg/ml for ofloxacin, 8 μg/ml for nalidixic-acid, and 1 μg/ml for cefixime; the zone of inhibition 19 mm, ≤ 28 mm, 17 mm, 19 mm, 19 mm for respective antibiotics was attained.

### Indicators of NARS with FQs

**Fig. 2:**
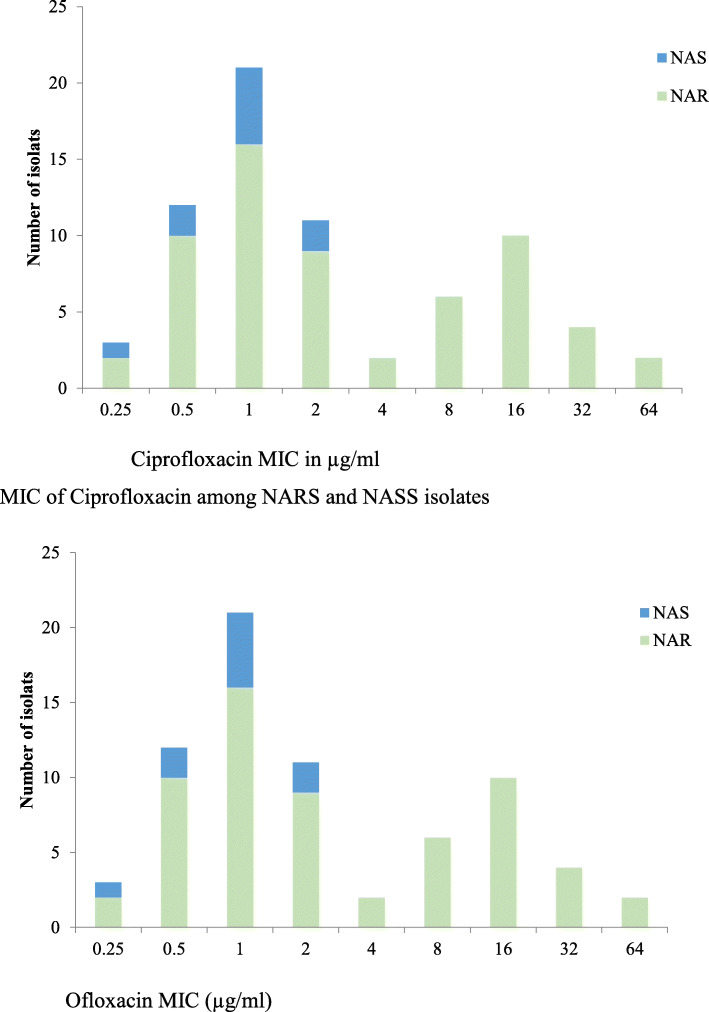
MICs of FQs (ciprofloxacin and ofloxacin) among NARS and NASS isolate

The MICs of FQs (ciprofloxacin and ofloxacin) among NARS and NASS isolates are shown in Fig. 2. The scatter-plot correlating the MICs of ciprofloxacin, ofloxacin, and nalidixic-acid against *Salmonella* isolates are shown in the (supplemental fig. 1 and fig. 2). The plot reveals a simultaneous presence of reduced FQs susceptibility in NARS.

## Discussion

The enteric fever or typhoid is one of the leading diagnosis of febrile illness in Nepal—a series of outbreaks with varying antimicrobial resistance trends have been reported [21–23]. Limited epidemiologic data with age categories up to 14 years are available; nevertheless, a pocket endemic region [24]. In these perspectives, estimation of the disease burden and its etiologies along with antimicrobial susceptibilities trend are obligatory factors requiring to effective prevention and control interventions.

The infection links strongly with low socio-economic status and unsanitary living conditions. Most of the urban areas of South Asia with growing population density and those lacking accessible safe water and food had sought as the high reservoirs [2, 3, 8, 25]. The highest incidence of salmonellosis up to 15.6% was recorded, in Nepal, including all age categories [26]. In pediatric population, we reported 4.9% culture-confirmed cases of * Salmonella *spp. which is more than twice the reported 2.0% by Prajapati et al. in 2008 [27]. The increasing rate could be due to surging population densities and lacking hygienic food/water supply in Kathmandu. Besides, a discrepancy in sample volume collected (particularly in infants and small kids where the required volume could not be drawn) as requires for culture could be one probable reason for the low rates of culture-positive enteric fever. Additionally, self-medication prior to hospital arrival (often practiced in rural areas of Nepal) could be another factor contributing to the low culture-positivity rate. These antibiotics might have lowered the bacterial load (requiring to grow) but not had eradicated it. In our study, we noted many self-medicated cases with FQs.

While relating the age categories, the higher incidence of enteric fever was observed in age-group 5to 9 years (primary school-going children) in our study. The probable reason could be outside tiffin habits in local dhaba or hotels, where handwashing facilities are not available—even foods are also rarely fresh.

Turning to the epidemiological scenario, *Salmonella* Typhi (S.Typhi) is the causative agent of nearly 30% of community-acquired febrile illness in Asia and 10% in Africa; but in Nepal, India, and China the predominance etiology contributing enteric fever is *Salmonella* Paratyphi (S.Paratyphi) [3, 7]. In our study, out of 90 culture-confirmed cases of salmonellosis, 62(68.8%) were caused by S. Typhi and the remaining 28 (31.1%) were S. Paratyphi A. The predominance of serovar Typhi was found as per the observations made by Zellweger et al.68.5% and 30.5% and Petersial et al. 55.7% and 44.3% respectively for serovars S. Typhi and S. Paratyphi [24, 25]. Shirakawa et al., nevertheless, reported S. Paratyphi as a more prevalent serovar [28]; his finding is corresponding to Pramod et al. (35.9% S. Typhi and 64.1% S. Paratyphi) [29]. There is no such well-established reason behind this variation of serovars; however, it can be assumed, the higher incidence of Typhi could be achieved via water-borne transmission as requires smaller inocula than paratyphoid which requires larger inocula via a food-borne transmission [23]. Furthermore, the self-medicated cases with FQ, in our study might result in a lower rate of S. Paratyphi A cases.

FQs and nalidixic-acid are the most prescribed antibiotics against salmonellosis in low-income-countries due to their cost-effectivity, easy accessibility (even sold from the medical pharmacies without a prescription), and availability in oral forms [7, 24, 25, 30]. With the emergence of NARS strains, globally; however, their efficacy against enteric fever is now questionable [31, 32]. It has been assumed that due to mutation in the genes coding for DNA gyrase (gyrA and gyrB) and topoisomerase IV (parC and parE), a high level of nalidixic-acid resistance occurs [33]. Similarly, lower susceptibility to fluoroquinolones possibly occurs due to the enhanced active efflux and early overproduction of the AcrA pump in isolates with the gyrA mutation [33]. Turning to our study, 304 cases (218 on ciprofloxacin; 86 on ofloxacin) had self-medicated history (treated with-out knowing etiologies and drug resistance pattern); despite, this resistance trend. We observed very high rates of FQs and nalidixic-acid resistance, but relatively low rates of resistance to first-line drugs. The findings are in line with recent epidemiological studies conducted in the Nepalese population [22, 24, 25, 27]. Possibly, due to the discontinuation of the commonly used antibiotics, of these days, in the therapeutic regimen for a longer time, and possibly the high molecular weight self-transmissible plasmid inducing resistance could have lost or de novo susceptibility [22, 34].

In our study, among tested antibiotics, a low rate of azithromycin resistance was observed on MIC which is similar to Khanal et al. reported [34]. In the Nepalese population, treatment failure on azithromycin treatment is yet not reported; nevertheless, an increase in MIC was reported in the patients from other countries. Relying upon this background, we can advocate for its choice as empiric therapy against salmonellosis.

Additionally, the third-generation cephalosporins (ceftriaxone, cefotaxime, and cefixime) had shown excellent effectiveness against *Salmonella* serovars with sensitivity up to 100% [35, 36]. In our study, 98.8% of the isolates were sensitive against third-generation cephalosporins supporting their use as well.

Moreover, MDR *Salmonella* isolates with fluctuating resistance trends have been increasingly reported from Asian countries [21, 28, 37, 38]. In our study subjects, fortuitously, no MDR salmonella isolate was recovered though this has been reported earlier from Nepal [25].

### Limitation

Among self-medicated cases, FQ-sensitive isolates might have failed to grow contributing to the lower incidence; therefore, the exact incidence of the disease could be even greater than observed. Probably, this could be the major drawback of our study. Although, a hospital-based study, we could not evaluate the risk factors and treatment outcomes in our settings. Further, the clinical evaluations of a large study population in the multicentral health institutions, if could have possible, it would be more elucidative. Besides, lacking the molecular laboratory set-up (presumed as a necessity for high-quality data in clinical studies) in our settings was another drawback, since blood culture has limited sensitivity.

## Conclusion

Regardless of surging drug-resistant *Salmonella enterica* cases elsewhere, the level of resistance was not as high as predicted in our study population. MDR trends may vary, therefore drug susceptibility testing side-by-side to empirical therapy is mandatory—particularly in developing countries where there is a practice of self-medication. Referring to our findings, higher susceptibility of *Salmonella enterica* to the conventional anti-typhoidal drugs was attributed compared to macrolides and fluoroquinolons. Therefore, reconsideration of these antibiotics as implicated therapies could be useful in clinical management. However, the decreased susceptibility against fluoroquinolones, alone and, in nalidixic-acid-resistance strain negates its empirical use.

## Supplementary Information

**Additional file 1: Supplemental Figure 1.** Scatter plot relating ciprofloxacin MICs to nalidixic acid MICs and disk. **Supplemental Figure 2.** Scatter plot relating ofloxacin MICs to nalidixic acid MICs and disk.

## Data Availability

Data generated or analyzed during this study are included in this manuscript and the remaining are available from the corresponding author on reasonable request.

## Abbreviations

AMR: antimicrobial resistance
ASM: American Society for Microbiology
ATCC: American Type Culture Collection
CLSI: Clinical and Laboratory Standard Institute
MDR: multiple-drug resistant
NARS: nalidixic-acid resistance Salmonella
NASS: nalidixic-acid sensitive Salmonella
S. Typhi: Salmonella Typhi
S. Paratyphi: Salmonella Paratyphi; ZOI: zone of inhibition
