# Supplementary material for: Susceptibility pattern of Salmonella enterica against commonly prescribed antibiotics, to febrile-pediatric cases, in low-income countries
Source: BMC Pediatr. 2021 Jan 15;21:38. doi: 10.1186/s12887-021-02497-3 (PMC7809854; doi:10.1186/s12887-021-02497-3)
Supplement: Supplementary file 1 — Additional file 1: Supplemental Figure 1. Scatter plot relating ciprofloxacin MICs to nalidixic acid MICs and disk. Supplemental Figure 2. Scatter plot relating ofloxacin MICs to nalidixic acid MICs and disk. [file 12887_2021_2497_MOESM1_ESM.docx]

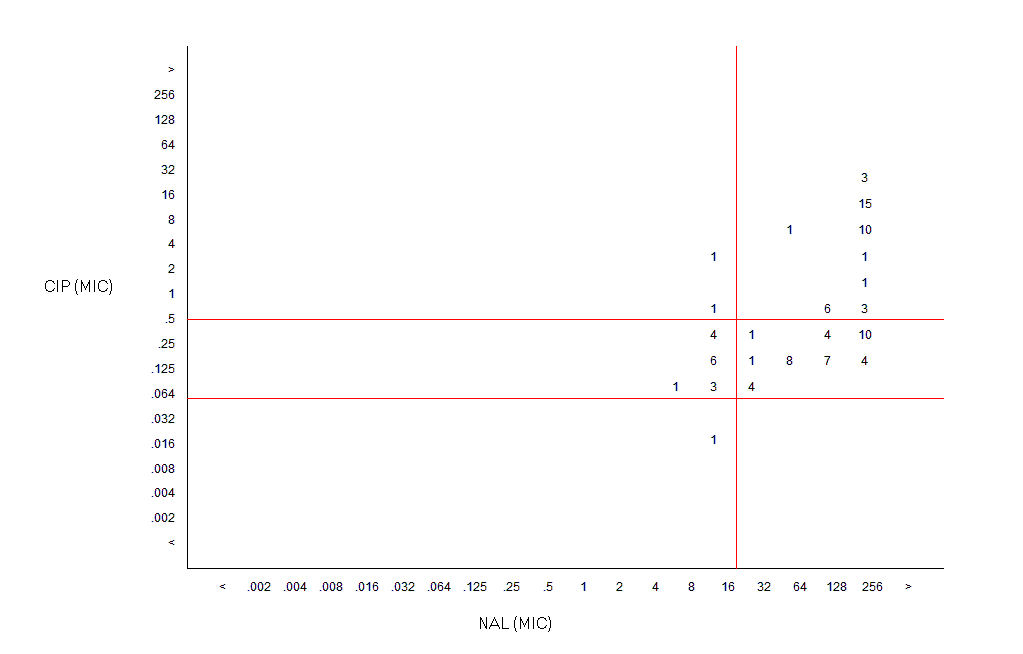


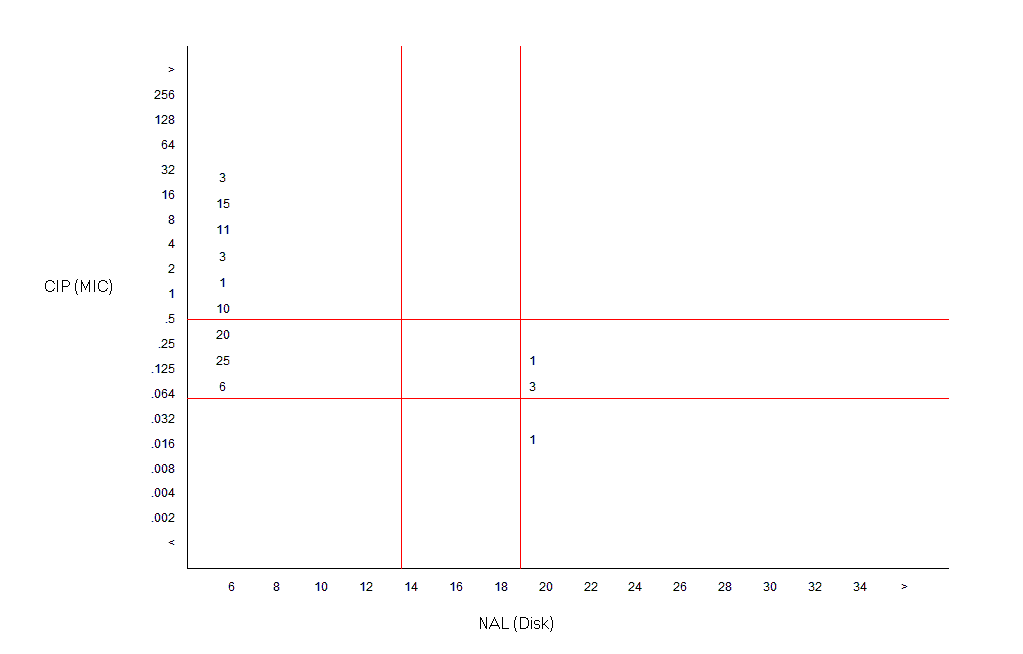


Supplemental Figure-1: Scatter plot relating ciprofloxacin MICs to nalidixic acid MICs and disk.


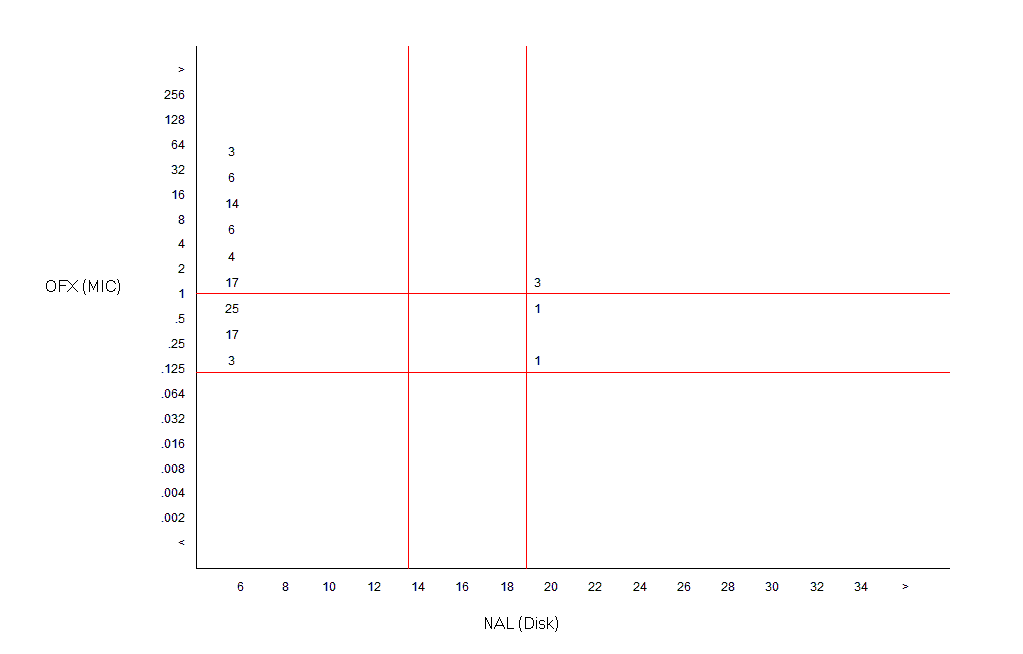


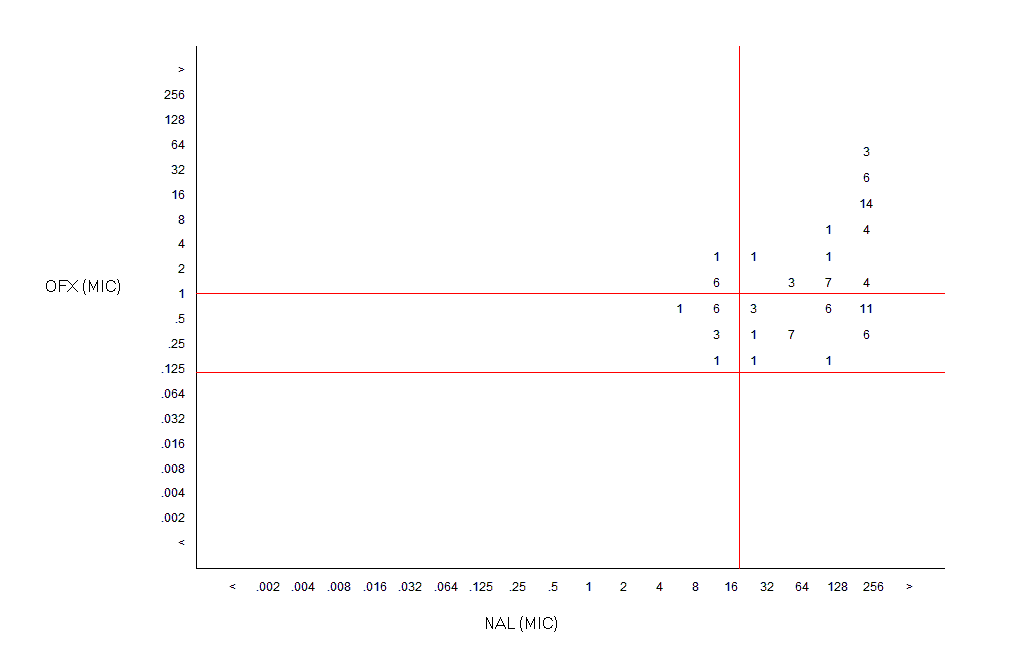


Supplemental Figure-2: Scatter plot relating ofloxacin MICs to nalidixic acid MICs and disk.
